# Assessing excess mortality in times of pandemics based on principal component analysis of weekly mortality data—the case of COVID-19

**DOI:** 10.1186/s41118-021-00123-9

**Published:** 2021-08-09

**Authors:** Patrizio Vanella, Ugofilippo Basellini, Berit Lange

**Affiliations:** 1 Department of Epidemiology, Helmholtz Centre for Infection Research (HZI), Inhoffenstr. 7, DE-38124 Brunswick, Germany; 2 Chair of Empirical Methods in Social Science and Demography, University of Rostock, Ulmenstr. 69, DE-18057 Rostock, Germany; 3 Laboratory of Digital and Computational Demography, Max Planck Institute for Demographic Research (MPIDR), Konrad-Zuse-Str. 1, DE-18057 Rostock, Germany; 4 Institut National d’Etudes Démographiques (INED), 9 cours des Humanités, FR-93322 Aubervilliers, Cedex, France; 5 German Center for Infection Research (DZIF), Inhoffenstr. 7, DE-38124 Brunswick, Germany

**Keywords:** COVID-19 pandemic, Excess mortality assessment, Mortality forecasting, Cross-country mortality trends, Principal component analysis, Time series analysis, Monte Carlo simulation, Stochasticity, Demography, Epidemiology

## Abstract

The COVID-19 outbreak has called for renewed attention to the need for sound statistical analyses to monitor mortality patterns and trends over time. Excess mortality has been suggested as the most appropriate indicator to measure the overall burden of the pandemic in terms of mortality. As such, excess mortality has received considerable interest since the outbreak of COVID-19 began.

Previous approaches to estimate excess mortality are somewhat limited, as they do not include sufficiently long-term trends, correlations among different demographic and geographic groups, or autocorrelations in the mortality time series. This might lead to biased estimates of excess mortality, as random mortality fluctuations may be misinterpreted as excess mortality.

We propose a novel approach that overcomes the named limitations and draws a more realistic picture of excess mortality. Our approach is based on an established forecasting model that is used in demography, namely, the Lee-Carter model. We illustrate our approach by using the weekly age- and sex-specific mortality data for 19 countries and the current COVID-19 pandemic as a case study. Our findings show evidence of considerable excess mortality during 2020 in Europe, which affects different countries, age, and sex groups heterogeneously. Our proposed model can be applied to future pandemics as well as to monitor excess mortality from specific causes of death.

## Introduction

The outbreak of COVID-19 has highlighted the need for sound and timely statistical analyses and monitoring of mortality patterns and trends. On many occasions, excess mortality, which is the number of deaths above expectations in the absence of exceptional events, e.g., a pandemic, exceptional influenza season, or heatwave, is considered to be the most appropriate indicator to measure the overall burden of the pandemic with respect to mortality (National Academies of Sciences & Medicine, 2020). As such, the excess mortality due to COVID-19 has recently received considerable attention, including tracking by major news outlets of this indicator across countries (see, e.g., The Economist, 2020). The COVID-19 pandemic has stimulated the demand for the timely release and publication of mortality data by national authorities (Leon et al., 2020). While aggregate all-cause mortality data are increasingly being released, the timely reporting of cause-specific data by demographic subgroups is still underdeveloped. However, such information would enable near real-time assessments of the excess mortality caused by specific diseases. Moreover, the widespread statistical approaches used thus far for estimating excess mortality have been rather simplistic, as they rely on rather basic statistical measures, such as the number of deaths above an ex ante expected value, which do not include or consider the stochasticity or connections among the mortality developments of different demographic or geographic groups. Strong correlations in mortality trends exist not only among different demographic groups but also among adjacent countries (Vanella, 2017); the underlying factors that drive mortality reductions, such as advances in medical care and hygiene, reach all of these groups to some extent (Vanella & Deschermeier, 2020). Therefore, advanced excess mortality assessments should simultaneously analyze a multitude of demographic and geographic groups. Furthermore, rather short time series are generally considered in the computations of excess mortality, which cannot sufficiently capture long-term trends.

In this article, we propose a stochastic framework for estimating excess mortality that is based on a Lee-Carter modeling approach. We develop a comprehensive model that can consider the multidimensionality (and eventual collinearity) of the data analyzed, which consist of several long-term time series for 19 different countries, both sexes, and four age groups. This allows us to consider the long-term mortality trends present in the data. Finally, the model can produce probabilistic statements concerning the excess mortality that occurs during a particular event. While our case study addresses the current COVID-19 pandemic, our method can be applied to future outbreaks of other pathogens, as well as to other major events that influence mortality at larger demographic and/or geographic scales.

The following section provides a literature review starting with the current approaches used for excess mortality estimations in general and specifically during the COVID-19 pandemic. Then, we provide an overview of stochastic mortality models, which address multiple populations in parallel. The latter provides a theoretical basis for our multidimensional mortality forecast model, which is presented in Section 3. Based on this, a stochastic investigation of excess mortality during the COVID-19 pandemic by country and demographic group is conducted, and the results are presented in Section 4. These results are then discussed, along with their implications for mortality forecasting. We finally draw conclusions from our findings and provide an outlook on the further need for developments in excess mortality evaluations and mortality forecasting.

## Literature review

### Assessment of excess mortality and estimates for COVID-19

Excess mortality due to certain circumstances is not directly observable. We would need to know how many deaths would have been observed without the event under study, which in our case, is the COVID-19 pandemic. Therefore, we compare the observations to a hypothetical alternative scenario in which the event supposedly causing excess mortality had not occurred. The outcome under this scenario can only be estimated based on modeling. We can estimate this hypothetical course based on forecasting by using historical data as a baseline and extrapolating the mortality trends from the data. In our case study, we estimate how many deaths would have occurred in 2020 if no pandemic or measures against the pandemic had occurred. These hypothetical deaths from the mortality forecast can then be compared to the observed deaths to quantify the amount of excess mortality due to the pandemic.

Estimations of excess mortality date back to the studies on influenza and pneumonia conducted by Collins et al. for the USA (Collins, 1932; Collins, Frost, Gover, & Sydenstricker, 1930). The authors calculated the weekly expected death rates due to influenza, pneumonia, and other causes for the whole population^1^ as the median of a 7-year baseline period. These were then compared to the observed death rates due to these causes during a certain period of an epidemic. The positive differences between the observed and expected mortality rates were then defined as excess mortality. Serfling (1963) extended this approach by fitting parametric Fourier (i.e., trigonometric) models to death rate time series separately by age groups and by selected causes of death^2^ for estimating monthly excess mortality. Housworth and Langmuir (1974) proposed a stochastic extension of Serfling’s approach and assumed that the residuals between observed and expected death rates followed a t distribution. Foppa and Hossain (2008) proposed a Bayesian extension of that model for the excess death numbers due to influenza.

Some approaches have been proposed to estimate the excess mortality due to COVID-19 during the current pandemic. We present some already published results in the form of scientific publications and official reports here.

Magnani et al. estimated the expected mortality rates and daily death numbers for Italian regions from January 1st to April 15th, 2020, by averaging the daily mortality rates for the years 2015–2019. Assuming that the death counts follow a Poisson distribution, they also estimated the 95% confidence intervals (CIs) for the daily death numbers separately for the age groups below 60 years and 60 years and above. They derived a statistically significant excess mortality in Italy due to COVID-19 from March 7th until the end of the study period and estimated 45,032 mean excess deaths overall, a figure that is more than double the death numbers officially attributed to COVID-19 (Magnani, Azzolina, Gallo, Ferrante, & Gregori, 2020). This discrepancy is likely due to an undercounting of COVID-19 deaths in the statistics, deaths due to other causes indirectly associated with COVID-19, or a flawed forecasting procedure used for quantifying the expected deaths. Michelozzi et al. (2020) further showed that this excess mortality was concentrated in northern Italy, which has been hit harder by the pandemic than in central and southern Italy, and that excess mortality is more prevalent in men and the elderly.

The New York City Department of Health and Mental Hygiene (DOHMH) COVID-19 Response team used the regression model of the US Centers for Disease Control and Prevention (CDC) based on the years 2015–2019 to estimate the number of expected deaths. The differences between the observed numbers of deaths and the ex ante expectations were defined as excess deaths. This surveillance system is normally applied to estimate the excess deaths that are attributable to influenza (Centers for Disease Control and Prevention, 2019), but it was also used to estimate the excess deaths due to COVID-19 in New York City between March 11th and May 2nd, 2020. The authors identified over 24 thousand excess deaths over the observed period, of which close to 14 thousand were laboratory-confirmed COVID deaths, while the other 5 thousand were probably associated with COVID-19 (New York City Department of Health and Mental Hygiene COVID-19 Response Team, 2020).

The Institute for Health Metrics and Evaluation (IHME, 2021) recently presented an ensemble approach for estimating the excess death rates for 2020, which estimates the total COVID-19-associated death rate based on the expected weekly or monthly death rates derived from past data, observed overall death numbers, and reported COVID-19 deaths. The model aims to derive the actual COVID-19-associated deaths to account for inaccurate national reporting for the 20 study countries. EUROMOMO provides concurrent excess mortality estimates based on a fit generalized linear model (GLM) by age group for 23 European countries. The model is fitted to a maximum of five previous years of data. The graphs reported by EUROMOMO show a general pattern of excess mortality in Europe since April 2020 for individuals aged 15 and older (Statens Serum Institut, 2020a, 2020b).

These approaches do not include the correlations of mortality rates among age groups and/or among countries. From a cross-country perspective, it would be appropriate to include these. The mortality trends in Europe share strong common patterns, not only among demographic groups but also among countries (Bergeron-Boucher, Canudas-Romo, Pascariu, & Lindahl-Jacobsen, 2018; Vanella, 2017), as the underlying factors causing mortality decline, such as medical advances, better hygiene, economic and educational advances, or better nutritional behavior, tend to affect different demographic and adjacent geographic groups simultaneously (Luy & Di Giulio, 2006; Vanella & Deschermeier, 2020; World Health Organization, 2015). Similarly, we observe how a pandemic affects the mortality levels of different groups at the same time (Statens Serum Institut, 2020a). Disregarding these correlations in the analysis would ignore these concurrent mortality developments, which would lead to biased prediction intervals in the forecasts.

Furthermore, the aforementioned approaches do not consider long time series of weekly or monthly mortality data, which is an important factor given the observed long-term trends of mortality improvements. The cardiovascular revolution that started around the 1970s in Europe provided strong improvements in mortality, especially at older ages (Vallin & Meslé, 2004; Vaupel et al., 1998). Not including these trends in the analysis might lead to a systematic bias in excess mortality estimations. Our proposed approach aims to overcome these limitations by employing a demographic perspective. Appendix A provides a summary comparison of the presented approaches and results, including ours.

More recently, other approaches to estimate excess mortality levels across countries have been proposed. Kontis et al. (2020) introduced an ensemble of 16 Bayesian models to estimate the excess mortalities in 21 industrialized countries during the first wave of the COVID-19 pandemic. Nemeth, Jdanov, and Shkolnikov (2021) implemented six different approaches to estimate the baseline mortalities for all countries in the short-term mortality fluctuations dataset of the Human Mortality Database and introduced a web-based application for visualizing the excess mortalities across age groups, years, and countries. Finally, Islam et al. (2021) estimated the excess mortalities in 29 high-income countries during 2020 by employing an overdispersed Poisson regression model.

### Multi-population stochastic mortality forecasting

There is a large amount of literature on mortality forecasting approaches. As it is not our intention to provide a full literature review here, interested readers are referred to the compilation by Janssen (2018). We will restrict our review to those approaches that we believe are important in this context, which are stochastic models that include age-specific mortalities and multiple populations.

One forecast approach, which is of major importance, is based on principal components (PCs). A PC is a linear combination of a group of variables and in our context, age-specific mortality rates. The PCs are derived by singular value decomposition. This method has two major advantages. First, the high dimensionality, which results from a collection of several mortality rate variables among age groups, sex, and countries, can be analyzed relatively efficiently. Second, the correlations among different variables, such as age- and sex-specific mortality rates, are included in the analysis, which are very important in forecasting to adequately quantify the uncertainties of mortality forecasts. An illustrative explanation of the method when applied to age- and sex-specific survival rates is given by Vanella (2018). The application of principal component analysis (PCA) to age-specific mortality rates goes back to Ledermann and Breas (1959), who used it for transforming French data to derive common mortality trends. Le Bras and Tapinos (1979) proposed the use of PCA to project mortalities in France. Bell and Monsell (1991) extended this framework by including autocorrelations of the PCs by employing autoregressive integrated moving average (ARIMA) models^3^ for mortality forecasting in the US. Lee and Carter (1992) identified the first PC in that model as a general mortality index, which covered the vast majority of mortality trends that were observed over all age groups and proposed a random walk with drift model to forecast the index, which can then be retransformed to forecast mortality rates.

Tuljapurkar, Li, and Boe (2000) qualitatively showed that there were large correlations in mortality trends among the G7 countries, which could be covered well by the Lee-Carter model. Booth, Maindonald, and Smith (2002) proposed a graphical method for determining the optimal baseline period to inform the model. A baseline that is too short assumes that the long-term future follows the near past, which appears to be unrealistic. On the other hand, the very long past data may not apply to future trends, especially in the shorter term. While the mortality index is modeled as a linear process, Brouhns, Denuit, and Vermunt (2002) proposed a GLM version of the Lee-Carter model. The classic Lee-Carter model assumes independence between the mortalities of females and males, which can be rejected (see, e.g., Bergeron-Boucher et al., 2018; Vanella, 2017, on the correlation of mortality among both sexes). Li and Lee (2005) therefore proposed an extension, the so-called common factor model, which includes the correlations in cross-country mortality and the correlations between the two sexes in the mortality trends to some degree in the analysis. Hyndman and Ullah (2007) proposed a nonparametric extension of the Lee-Carter model. Russolillo, Giordano, and Haberman (2011) proposed extending the Lee-Carter model by applying a three-mode PCA to include the correlations in cross-country mortalities in the model. However, they ignored the sex-specific differences in their model. Vanella (2017) proposed a simulation approach that forecasts age- and sex-specific survival rates for 18 European countries while considering the correlations in mortality trends among age groups, sexes, and countries via PCA. The author demonstrated an efficient way to include similar mortality developments among different countries in one model, as PCA can cover the majority of the trends that are witnessed simultaneously by different countries. We will use a derivation of that approach for our analysis. Bergeron-Boucher, Canudas-Romo, Oeppen, and Vaupel (2017) proposed a modification of the Li-Lee model by leveraging age-at-death distributions and compositional data analysis to produce coherent forecasts for 15 Western European countries.

From our literature review, we see that, with a few exceptions, research on mortality forecasting has focused on a national level. In some cases, the mortality forecasts for a collection of countries, or even at the global scale, are of interest. Separate forecasts would not only be unfeasible but would also ignore common trends among countries. Some authors have conducted stochastic projections of groups of countries or at a global scale by using Bayesian approaches, which assume an a priori distribution for some parameter or variable either based on auxiliary data or subjective assumptions (see Kruschke, 2015; Lynch, 2007 on Bayesian modeling). To capture the major problem of the Lee-Carter model of systematically underestimating the uncertainties in mortality forecasts, Pedroza (2006) proposed a Bayesian extension of the classic Lee-Carter model, which includes the uncertainty of all parameters by using a Markov chain Monte Carlo (MCMC) simulation. King and Soneji (2011) suggested considering the assumptions of the trends in smoking behavior and obesity in the projections of age-specific mortality rates for the US through a Bayesian hierarchical model. Raftery, Chunn, Gerland, and Ševčíková (2013) proposed a Bayesian hierarchical model for joint probabilistic projections of cross-country male life expectancies by cohort using time series data on life expectancy in combination with judgmental projection data by national experts. The approach was then expanded for females by simulating the gender gap in life expectancy by regression analysis of the international data (Raftery, Lalić, & Gerland, 2014). The Raftery model forms the basis of the life expectancy projections of the United Nations. From these projections, they derive age- and sex-specific mortality rates for all countries with three different techniques, which depend on the quality of the mortality data available for the countries under study (United Nations, 2019). Antonio, Bardoutsos, and Ouburg (2015) provided a Bayesian version of the Lee-Carter model that enabled joint mortality projections among various countries.

This review shows that there is a large battery of sophisticated approaches for stochastic and cross-country forecasting of mortality, which could be applied to provide more sophisticated and realistic estimations of excess mortality. However, the widely used approaches for excess mortality modeling do not make use of these possibilities thus far. Our contribution adds to the literature by improving the classic excess mortality estimations with modern methods in the stochastic forecasting of cross-country mortality.

## Data and methods

### Data

We extracted recently published estimates of weekly mortality rates by sex and age groups below 15 years, 15 to 64 years, 65 to 74 years, 75 to 84 years, and aged 85 and above, which were provided by the short-term mortality fluctuation data series of the Human Mortality Database (2021) (HMD). The data provide 52 weekly estimates of the mortality rates for a series of calendar years and start from different country-specific time points. To retain consistency with the annual mortality rates, the mortality rate of country *c*, gender *g*, age group *a*, in calendar week *w* in year *y*, *m*_*c*, *g*, *a*, *w*, *y*_, is calculated by dividing the death numbers of the demographic, geographic, and temporal combinations by the corresponding annual population exposure to the risk of death, *E*_*c*, *g*, *a*, *w*, *y*_, divided by 52:

$$ {m}_{c,g,a,w,y}=\frac{D_{c,g,a,w,y}}{\left({E}_{c,g,a,y}/52\right)} $$

We select all countries with available data since the start of 2000^4^. We take the data for the entire period from week 2, 2000^5^ to week 52, 2019 for Austria, Belgium, Estonia, Finland, France, Hungary, Israel, Latvia, Lithuania, the Netherlands, Norway, Poland, Portugal, Scotland, Slovakia, Slovenia, Spain, Sweden, and Switzerland, for which data are available and the population and death numbers are sufficiently high to derive representative estimates of weekly mortality levels. To avoid zero values in the data, we aggregate the age groups below 15 and 15–64 years into a single group. This is a very large group with heterogeneous mortality risk (Bohk-Ewald & Rau, 2017), especially concerning its fatality risk with respect to COVID-19 (Goldstein & Lee, 2020; Vanella et al., 2021). Alternatively, we could consider discarding persons from the youngest age group from the analysis. To retain the full data available, we prefer the first option, which, as we will show, does not bias our understanding of excess mortality in 2020. We compute the mortality rates in this wider group using the aggregated deaths and exposures that were extracted from the HMD. Finally, we arranged the data in a 1039×152 matrix^6^ of the time series of weekly age-, sex-, and country-specific mortality rates (WASCSMRs). In Appendix B, we report all of the country-, sex-, and age-specific combinations that are analyzed in our paper. For the final step of our analysis, we use daily reported data on COVID-19-associated deaths by country, which are provided by the European Centre for Disease Prevention and Control (2021a) (ECDC).

### Methods

We follow Vanella (2017), which was presented in 2.2. To stabilize the variances in mortality rates, especially for older ages, we employ a logit transformation of the WASCSMRs (Vanella, 2017). We first perform PCA on the logit-WASCSMR time series such that we obtain a set of PCs with the *i*th PC being a linear combination of all logit-WASCSMRs:

$$ {p}_{i,w,y}=\sum \limits_{j=1}^{152}{\lambda}_{i,j}{\mu}_{c,g,a,w,y},i=1,\dots, 152, $$

where *μ*_*c*, *g*, *a*, *w*, *y*_ is the logit-WASCSMR of country *c*, sex *g* and age *a*, in calendar week *w* of year *y* and *λ*_*i*, *j*_ is the loading of the *j*th mortality variable on the *i*th PC.

**Fig. 1:**
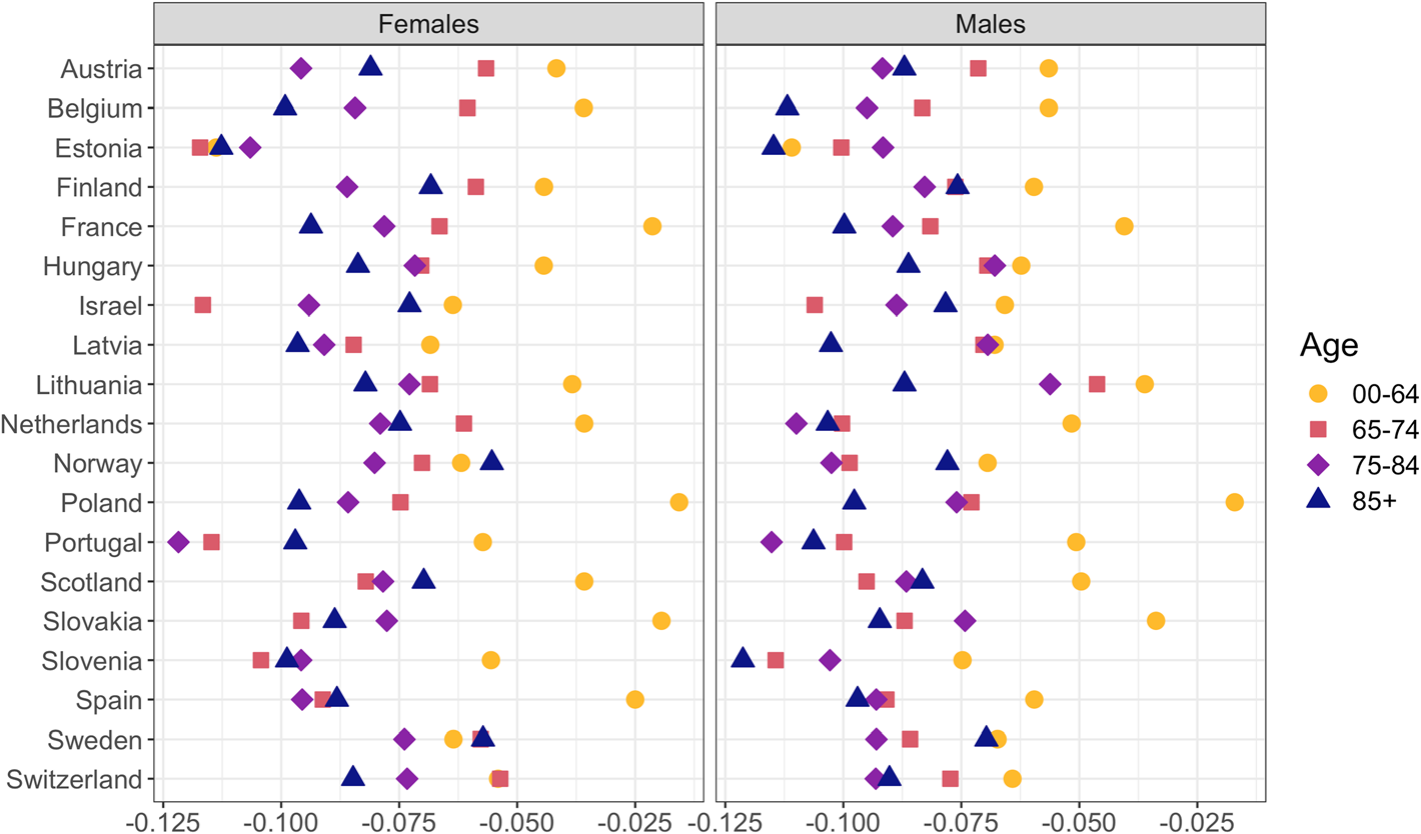
Loadings of principal component 1

Figure 1 shows the loadings of the first PC (PC1). The loadings can be interpreted as correlations between the PCs and original variables (Vanella, 2018) and in this case, the logit-WASCSMRs.

**Fig. 2:**
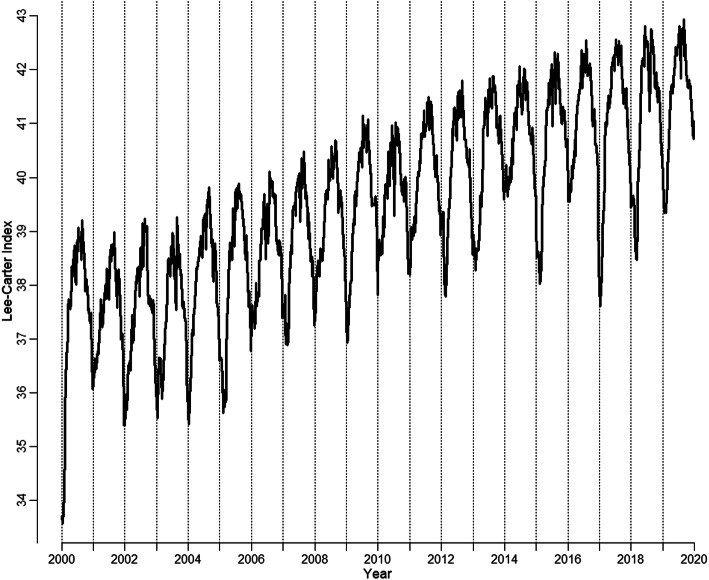
Past course of the Lee-Carter Index

The loadings of PC1 are strictly negative, which imply negative correlations with all mortality rates. Thus, increases in PC1 ceteris paribus are associated with decreases in all WASCSMRs under study. PC1 is hence a classic Lee-Carter mortality index (Lee & Carter, 1992) and explains 55% of the overall variance in the 152 time series. Therefore, we will refer to it as the *Lee-Carter Index* in the remainder of this paper. Furthermore, it is interesting to observe that the absolute values of the loadings for the younger age group (e.g., 0–64 years) are nearly always smaller than those for the older groups. As such, any increases in PC1 imply greater mortality reductions at older rather than younger ages. Figure 2 shows the time series of PC1 from 2000 to 2019. The vertical lines indicate week 1 of each year.

The curve exhibits a highly seasonal pattern, with strongly increasing mortality (i.e., lower PC1values) in the winter season and decreasing mortality in summer (i.e., higher values). The general trend is increasing, which corresponds to decreasing mortality trends but has been concave since approximately 2005, which means that mortality improvements have had a diminishing trend since then. To capture these different features of the time series, we iteratively fit models 1, 2, and 3 from Table 1 to the weekly PC1 values for the years from 2000 to 2019, as illustrated in Fig. 2, by using ordinary least squares (OLS). The three resulting models are compared via Akaike’s information criterion (AIC) and the Bayesian information criterion (BIC). Table 1 gives the results of this range of model fits.

**Table 1:** Iterative trend function coefficients with 95% CIs to Lee-Carter Index

Parameter	Model 1	Model 2	Model 3
**Intercept**	39.49 (39.4; 39.57)	33.4 (33.26; 33.54)	32.95 (32.79; 33.11)
$\mathbf{\cos}\left(\frac{\boldsymbol{\pi} \boldsymbol{w}}{\mathbf{26}}\right)$	1.34 (1.22; 1.47)	1.31 (1.27; 1.35)	1.05 (0.96; 1.15)
$\frac{\mathbf{\exp}\left(\frac{\boldsymbol{w}-{\boldsymbol{t}}_{\mathbf{0}}}{\boldsymbol{\beta}}\right)}{\mathbf{1}+\mathbf{\exp}\left(\frac{\boldsymbol{w}-{\boldsymbol{t}}_{\mathbf{0}}}{\boldsymbol{\beta}}\right)}$	-	9.73 (9.51; 9.95)	9.74 (9.53; 9.94)
**Spring**	-	-	0.71 (0.58; 0.83)
**Summer**	-	-	0.55 (0.36; 0.73)
**Autumn**	-	-	0.5 (0.4; 0.61)
***R***^***2***^	*0.3037*	*0.9153*	*0.928*
***AIC***	*3,707*	*1,520*	*1,358*
***BIC***	*3,722*	*1,539*	*1,392*

**Fig. 3:**
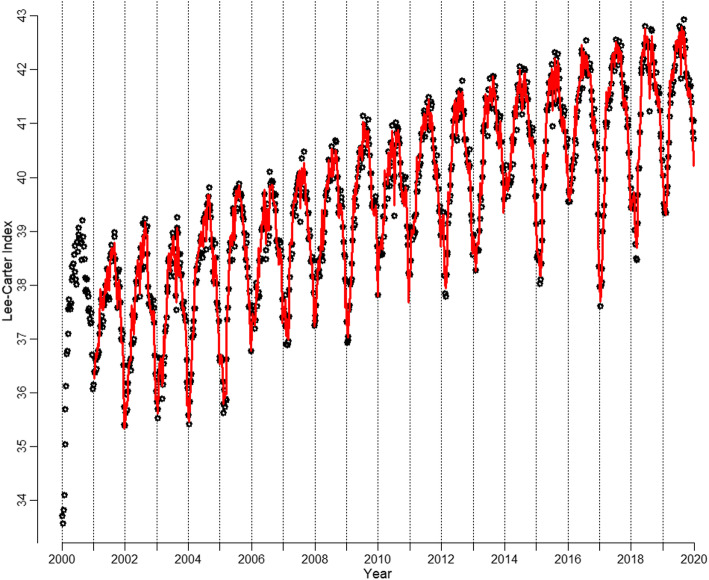
Course of the Lee-Carter Index for 2000–2019 with model fit

Model 1 includes a cosine term that represents the baseline seasonality of the year, which is similar to the Serfling approach, with *w* = 0 being calendar week 31, 2000. The transformation $\frac{2\pi }{52}=\frac{\pi }{26\ }$ of the argument leads to a periodicity of 52 weeks for the cosine term, as a standard cosine function has a periodicity of 2*π*. The interested reader may refer to Appendix C for more details on cosine functions. This choice of origin leads to the maximization of *R²*, which indicates the best fit to the observed seasonality. We checked the full Fourier model as well but discarded the sine term, as it does not lead to any improvement in the model fit while worsening the efficiency of the model, which is represented by higher values of the information criteria. Model 2 includes an inverse logistic growth function as a second explanatory variable, which can be used to simulate a growth function, which is similar to the implementations in Vanella (2017), Vanella and Deschermeier (2018), and Vanella and Deschermeier (2019), with *w* being the week and *t*_0_ being a parameter to be iteratively estimated to maximize the model’s *R²*. *β* is a parameter that was estimated by maximum likelihood before running the OLS^7^ regression, as it cannot be derived from OLS but instead must be defined beforehand. *Spring, Summer*, and *Autumn* in model 3 are binary variables, which are 1 during the respective seasons and 0 otherwise. Winter is therefore the baseline season. Spring runs from calendar weeks 13 to 25, summer runs from calendar weeks 26 to 38, and so on. By following Occam’s Razor, a simple model should be preferred to a more complex one if it performs similarly well (Bijak, 2011). A model is most efficient if it minimizes the information criteria. We see that the inclusion of an inverse logistic growth function in model 2 substantially increases the quality of the model in comparison to model 1, as it leads not only to a very large increase in *R*² from 30.4 to 91.5% but also to a large decrease in both the AIC and BIC. Therefore, the model fit to the data increases significantly, which leads to a more efficient model. However, this long-term trend is generally not considered in models of excess mortality, as explained in Section 2.1. An extension of the model by seasonal dummies, as in model 3, leads to additional significant improvements in the fit, as the trigonometric function systematically underestimates the mortality peaks in winter while overestimating the values in summer. Moreover, a cosine function is section-wise point symmetric, as illustrated in Appendix C. As the mortality trends during the year do not behave this symmetrically, the seasonal dummies provide some of the asymmetric behavior of the mortality curve, which is more realistic. Model 3 fits the data well with an *R*² value of nearly 93%. Both information criteria favor this model as well. The coefficients of the seasonal dummies should not be used for concrete interpretations, however. They simply serve as correction factors to systematic under/over-estimation of the Fourier series, which systematically underestimates mortality in winter and overestimates mortality in summer. The residuals from the quantified model are fitted by using a seasonal autoregressive integrated moving average (SARIMA) model, which is chosen by using a series of tests following Vanella (2018). Figure 3 illustrates the fit of model 3 (continuous line) to the data (dots). Please note that the line starts at the beginning of 2001 since the model prediction needs 1-year lags, which are not yet available in 2000.

The forecast function according to our optimal model 3, which is illustrated as a red line, is

$$ {PC}_1(w)=32.95+1.05\cos \left(\frac{w\ast \pi }{26}\right)+9.74\frac{\exp \frac{w-220}{482.05}}{1+\exp \frac{w-220}{482.05}}+0.71f+0.55s+0.5a+\alpha (w), $$

with

- PC_1_(*w*) is the value of the first PC in week *w*;
- $$ \alpha \left(\mathrm{w}\right)=\alpha \left(\mathrm{w}-1\right)+0.16\alpha \left(\mathrm{w}-52\right)-0.16\alpha \left(\mathrm{w}-53\right)+\varepsilon \left(\mathrm{w}\right)-0.26\varepsilon \left(\mathrm{w}-1\right),\varepsilon \left(\mathrm{w}\right)\sim N\;\left(0;0{.32}^2\right); $$
- *w* = 0 corresponds to calendar week 31, 2000;
- *f* has a value of 1 in the spring weeks, i.e., calendar weeks 13–25, 0 otherwise;
- *s* has a value of 1 in the summer weeks, i.e., calendar weeks 26–38, 0 otherwise; and
- *a* has a value of 1 in the autumn weeks, i.e., calendar weeks 39–51, 0 otherwise.

**Fig. 4:**
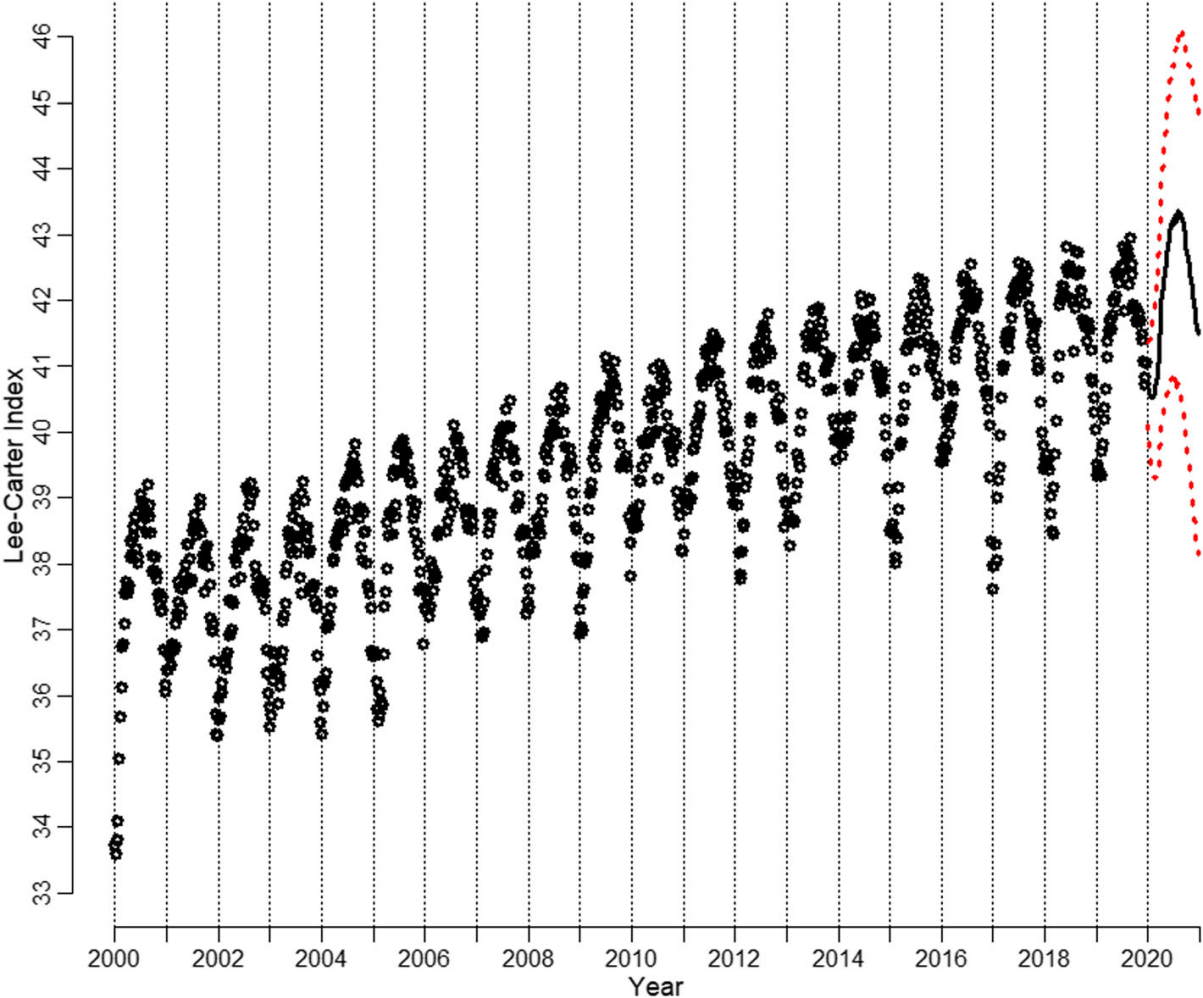
Historic course of the Lee-Carter Index with median forecast and 95% prediction intervals

Figure 4 shows the time series with the median forecast from model 3 with theoretical 95% prediction intervals (PIs).

The Lee-Carter Index can serve as a summary indicator of overall mortality, as it captures the main mortality trends^8^. The loadings of the remaining PCs are not considered to provide straightforward interpretations and will be assumed to be random walk processes^9^, following Vanella (2017), as our tests show that random walk models perform reasonably well in reproducing the series.

The Lee-Carter Index covers the general mortality trends among age groups, sexes, and countries (Lee & Carter, 1992; Vanella, 2017). Therefore, the weekly differences between its development and our forecast provide a general assessment of excess mortality by group. For this, we multiply the loadings from Fig. 1 by the HMD estimates of the WASCSMR for the year 2020 for the study countries, and thereby derive hypothetical observations of the Lee-Carter Index, and fix the loadings that were derived from the baseline data:

$$ {\hat{PC}}_1\left(\tau \right)={\sum}_{i=1}^{152}{\lambda}_{i,1}{\mu}_{i,\tau },\tau =1,2,\dots .,52, $$

with

- *λ*_*i*, 1_ is the loading of the *i*th WASCSMR on the Lee-Carter Index
- *μ*_*i*, *τ*_ is the HMD estimate of the *i*th logit-WASCSMR for week *τ*.

This will enable a direct comparison between the course of the PC and its expectation based on the time series data. The results of this approach will be presented in Section 4.

We then use a Monte Carlo simulation for each PC to simulate 10,000 trajectories of the weekly development of all PCs for 2020. Since the PCs are uncorrelated (Vanella, 2018), independent simulations of their future paths do not lead to biased estimations of mortality rates, which are then derived from these. The results consist of 10,000 trajectories of each PC, which can be retransformed into weekly trajectories of the logit-WASCSMR. For instance, let **Π**_*t*_ be the simulation matrix of all PCs (10,000×152) in period *t*. The corresponding simulation matrix of the logit-WASCSMRs is then

$$ logit\left({\boldsymbol{A}}_{\boldsymbol{t}}\right)={\sum}_{i=1}^{152}{\boldsymbol{\Pi}}_t{\boldsymbol{\Lambda}}^{-\mathbf{1}}, $$

where **Λ**^***−*1**^ is the inverse of the loading matrix that results from the singular value decomposition. In the next step, we derive the trajectories of the WASCSMRs by taking the inverse logit transform of *logit*(***A***_***t***_), namely, *logit*^−1^[*logit*(***A***_***t***_)] = ***A***_***t***_. All simulations of the mortality rates are now within the realistic range (0;1), which was achieved by the initial logit transformation of the input data.

The distribution of the differences among the observed WASCSMR and the respective forecasts can then provide a probabilistic statement regarding the actual degree of excess mortality that is observed during a certain period.

The last part of the analysis compares our weekly estimates of excess mortality with the officially reported COVID-19-attributed deaths to assess the differences between the two data sources. For this, we compute the excess mortality for the entire year of 2020, i.e., during the COVID-19 outbreak.

## Results

**Fig. 5:**
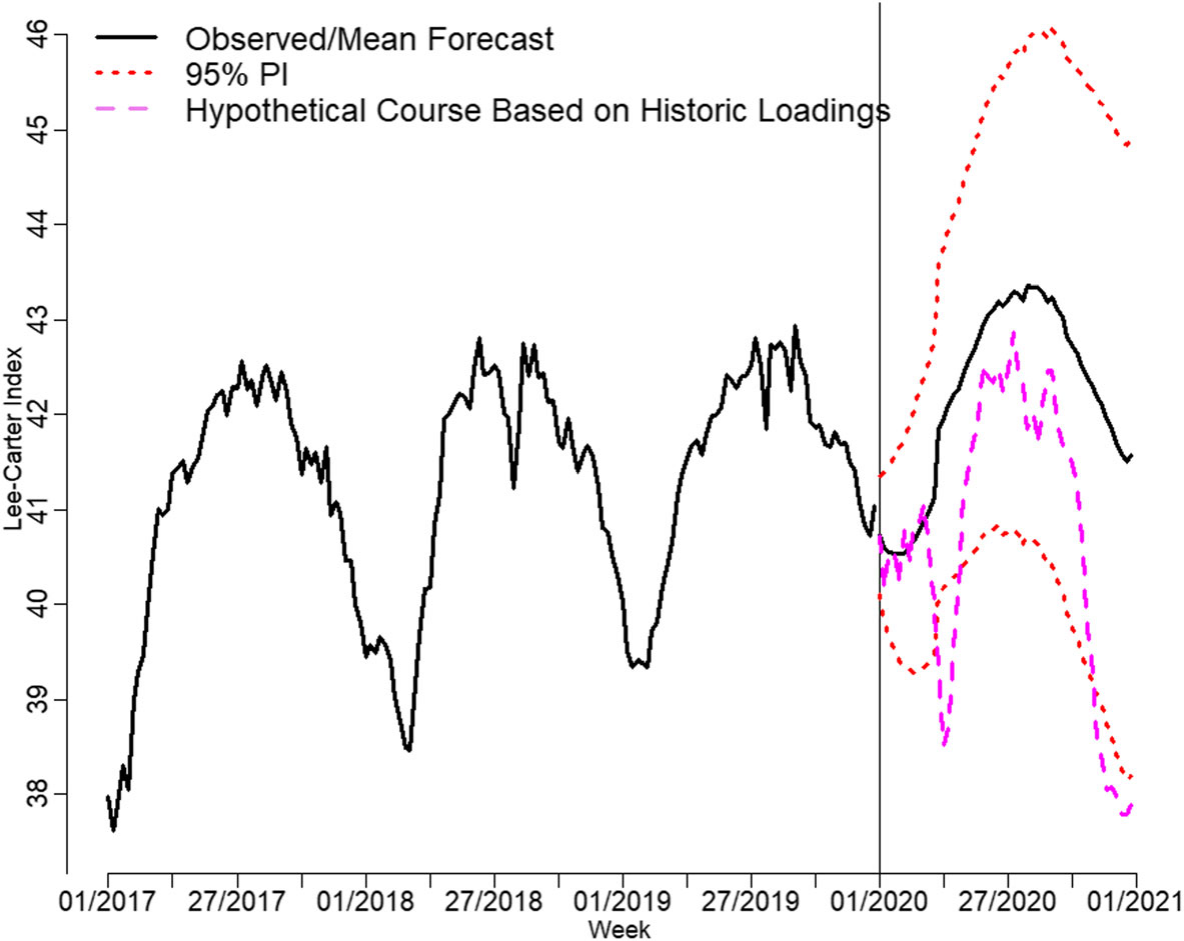
Forecast of Lee-Carter Index for 2020 with 95% PIs and actual course

Figure 5 shows the course of the Lee-Carter Index since the beginning of 2017 and its forecast until the end of 2020 with 95% PIs as described in Section 3. Moreover, the violet dashed line provides the hypothetical course under the loadings that were derived from the 2000–2019 data.

The mortality development oscillates around its mean forecast up to week 10, i.e., the first week of March 2020. After that, it leaves that course and sharply decreases. In week 13, it even falls below the lower bound of the 95% PI. Afterward, it stabilizes within the 95% PI, yet is below the expected course. In autumn 2020, the curve decreases sharply once more and exceeds the lower bound of the 95% PI. Overall, the Lee-Carter Index shows a level for 2020 that is similar to that in 2017. Thus, the cross-country mortality levels in early spring and autumn are statistically significantly higher than the realistic trends that were derived from the previous 20 years of data.

**Fig. 6:**
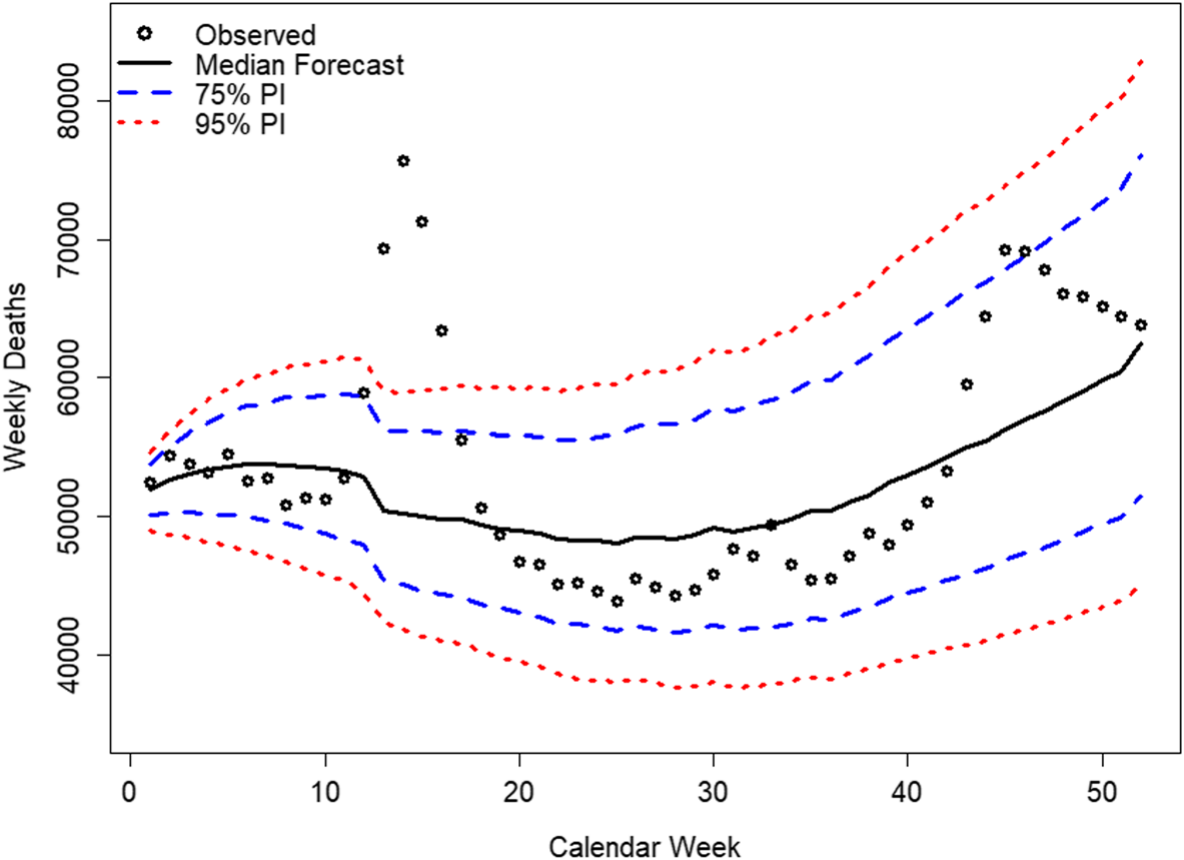
Observed and predicted weekly deaths in 2020 for the 19 study countries. Sources: Human Mortality Database (2021); Computations and design by the authors

By retransforming the PC forecast to forecasts of the WASCSMRs and multiplying those with the population estimates from the HMD, we derive weekly estimates of deaths for all subgroups, which enable comparisons of the observed mortality levels with the expected mortality levels in absolute numbers. Figure 6 illustrates the overall observed deaths for 2020 for the 19 study countries compared to the respective predictions with 75% and 95% PIs.

Most of the observations are within the limits of the 75% PI; in week 12, the upper limit is exceeded, while the dot remains within the 95% PI. Between weeks 13 and 16, however, the number of deaths exceeds the upper limit of the 95% PI. By the end of the year, the decrease in the Lee-Carter Index is mirrored by the increased death counts, which in calendar weeks 44 and 45 even exceed 75% PI. More detailed results, which are stratified by sex and age group, can be found in Appendix D.

**Fig. 7:**
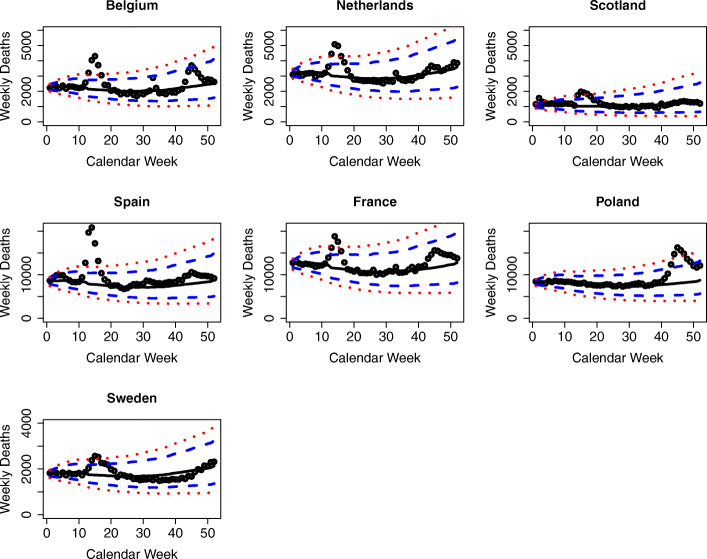
Observed and predicted weekly deaths in 2020 by country for countries with statistically significant excess mortality. Sources: Human Mortality Database (2021); Computations and design by the authors

**Fig. 8:**
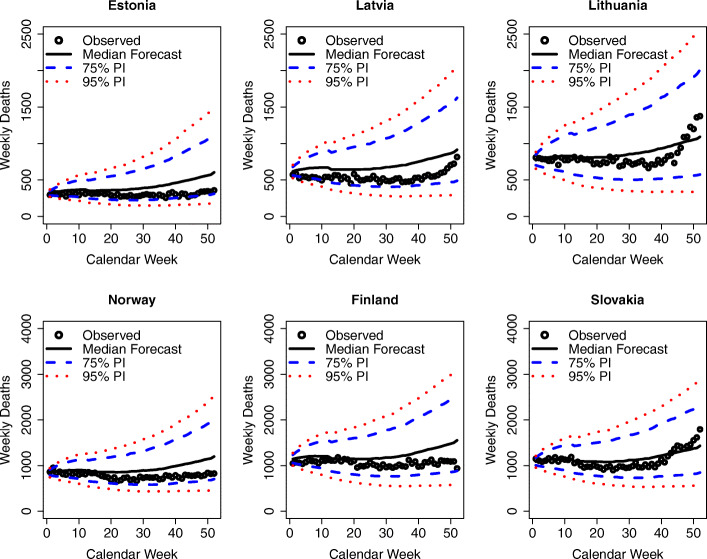
Observed and predicted weekly deaths in 2020 by country for the Northern European Countries without statistically significant excess mortalities. Sources: Human Mortality Database (2021); Computations and design by the authors

**Fig. 9:**
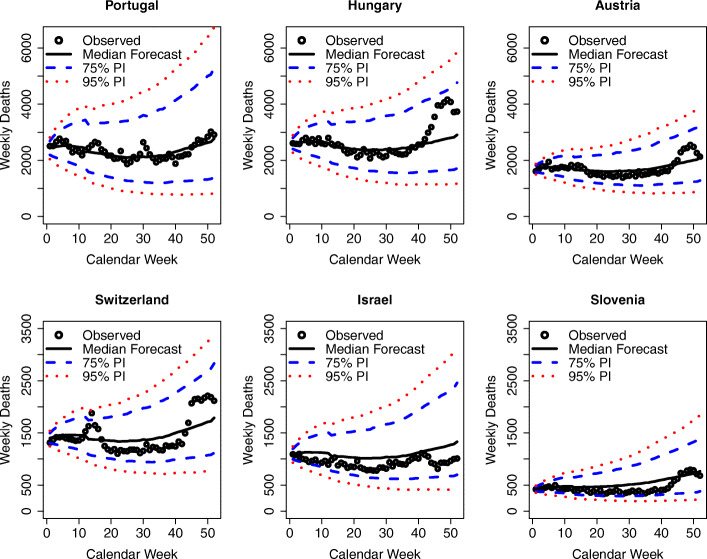
Observed and predicted weekly deaths in 2020 by country for the remaining countries without statistically significant excess mortalities. Sources: Human Mortality Database (2021); Computations and design by the authors

One limitation of analyzing aggregate results, such as those shown in Fig. 6, is that the 19 countries under study adopted different strategies to fight the COVID-19 pandemic, with some countries implementing stronger nonpharmaceutical interventions (NPIs) than others (European Centre for Disease Prevention and Control, 2021b). As such, more informative results can be derived from analyzing the country-specific results. Figures 7, 8, and 9 show the country-level excess mortality effects during the COVID-19 crisis. The countries shown in Fig. 7 exhibit significant excess mortalities, especially between calendar weeks 13 and 17. While the scales on the ordinate are the same for the countries located on the same horizontal axes of the panels, the reader should be careful with comparisons between the figures on one vertical axis, as the ordinates are different.

The case of Poland is special, where the deaths have been close to expectations for most of the year and only after week 40, in autumn 2020, exceeded the upper bounds of the PIs. This development has been suggested to be associated with the presidential vote in Poland taking place in summer 2020. In preparation for the vote, the Polish government loosened the strict COVID-19 response measures it had earlier established, as Poland appeared to have managed the epidemic well until then. Traveling was even subsidized, and pre-vote rallies were organized where many of the earlier measures were no longer adhered to. These changes in behavior ought to have been associated with sharp rises in infection rates and subsequent deaths observed since late summer (Kość, 2020).

Figure 8 shows the results for the Northern European countries, i.e., the Scandinavian and Balkan countries that were without significant excess mortalities.

These countries appear to have managed the epidemic well, and they not only show deaths that are close to expectations but are even mostly below expectations. In particular, the Balkan countries started proactive measures, such as border tests, in mid-January 2020 to avoid import of the virus by travelers (European Centre for Disease Prevention and Control, 2021b).

Figure 9 shows the analysis for the remaining study countries that had no significant excess mortalities. These include some Southern and Eastern European countries, which appear to have managed the epidemic well and kept the number of deaths remarkably close to the ex ante expectations or even below.

**Fig. 10:**
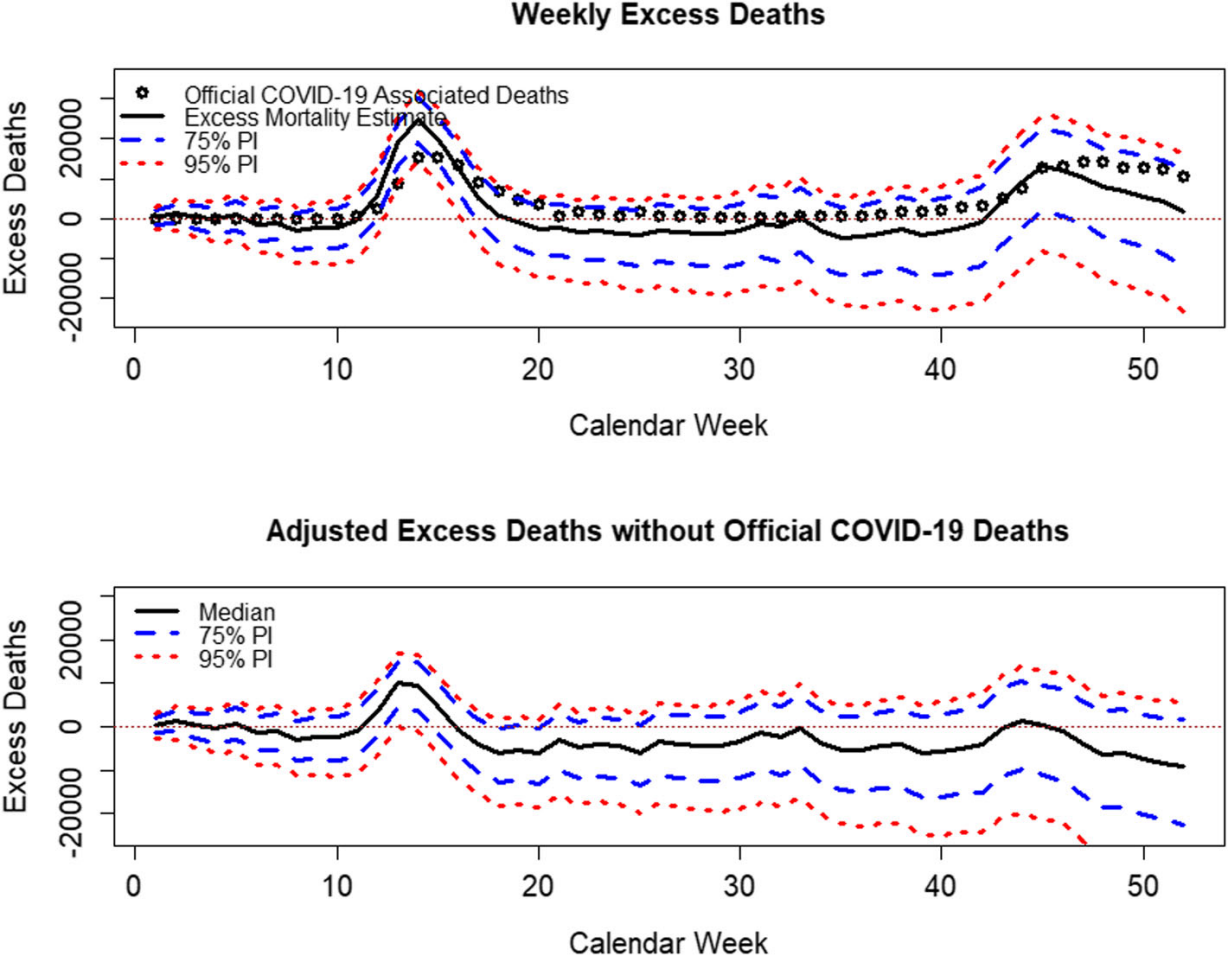
Excess mortality distribution with official COVID-19-associated deaths by calendar week for 18 study countries. Sources: European Centre for Disease Prevention and Control (2021a); Human Mortality Database (2021); Computations and design by the authors

Finally, we investigate how our results are related to the official data on COVID-19-associated deaths. Figure 10 shows the weekly excess mortality numbers for 18 of the study countries^10^, which are derived from our simulations with 75% and 95% PIs, along with the official COVID-19-associated deaths, as provided by the ECDC. The bottom panel shows the differences between the excess mortality estimates and COVID-19 deaths.

After subtracting the COVID-19 deaths, there were no significant deviations from the expected deaths until calendar week 12. The excess mortalities in calendar weeks 13 and 14 were slightly augmented even after adjusting for the COVID-19 numbers. After the introduction of COVID-19 countermeasures, the COVID-19 adjusted mortalities were below expectations for most of 2020, which indicates effective countermeasures; these countermeasures not only reduced the number of direct COVID-19 deaths but also showed a tendency to prevent deaths due to other causes. However, in autumn 2020, an increase in the number of excess deaths due to the second wave of COVID-19 occurs.

## Discussion

The COVID-19 pandemic has influenced the mortality patterns and trends across the world since its outbreak at the beginning of 2020. Similar to other analyses (Magnani et al., 2020; Michelozzi et al., 2020; Statens Serum Institut, 2020a), we confirm the presence of clear excess mortalities in several countries with strong infection dynamics during 2020. Since our study countries enacted strict countermeasures to contain the spread of the virus over the year, we found less evidence, with Poland as the exception, of statistically significant excess mortalities in the second half of the year. The death numbers, when adjusted for the official COVID-19-associated death statistics, even show slight tendencies to be lower during 2020 in comparison to their ex ante predicted levels, which implies that the NPIs introduced during 2020 not only reduced the number of direct COVID-19-associated mortalities but also decreased deaths due to other causes. However, preliminary studies have shown that the overall effect of the COVID-19 pandemic has had a large negative impact on life expectancies in most of the countries that we analyzed (Aburto et al., 2021).

Our estimate of excess mortalities is more precise than previous approaches and shows the uncertainty of these estimates that is based both on the demography of countries and long-term mortality trends. Previous approaches do not sufficiently include stochasticity in their predictions, as they neither consider autocorrelations of the mortality time series (be it death numbers or death rates) nor the cross-correlations among the mortality series in their models.

Moreover, some models do not consider the long-term trends in mortality at all, as they simply take the average values of the previous years. Our literature review has shown that there could be good arguments for considering a shorter baseline period in excess mortality estimations if we believe the longer baseline would not be representative for the near future. In such instances, the longer baseline could bias our predictions and lead to poor estimations of excess mortalities. This could be the case if we had observed extraordinary events in the long past, which we deemed irrelevant for predicting the near future. A baseline of 20 years, as we have chosen for our model, appears to be a good trade-off: the countries that we studied did not experience particularly acute circumstances during the baseline period, which would distort our excess mortality estimations. Moreover, we know from asymptotic theory that a longer baseline period, i.e., more input data, delivers better estimates of the variance (Wooldridge, 2013), and in our case, the predictions of future variances due to different mortality developments. Our models, especially the comparison between model 1 and model 2, have demonstrated the improvement in model fits by including a long-term trend. Some studies include trending behavior, but only for the last 4 or 5 years, which does not sufficiently cover the long-term mortality trend, as we observe decreasing mortality trends in the developed countries since at least the early 1970s (Vanella, 2017; Vaupel et al., 1998). Previous approaches to excess mortality estimation therefore systematically underestimate the variances in the forecasts.

Forecasts are less certain with increasing distance between the time at which the forecast was conducted and the time for which the forecast is conducted. This phenomenon is represented by the increasing widths of the PIs (e.g., Box, Jenkins, Reinsel, & Ljung, 2016; Vanella & Deschermeier, 2020). The literature on excess mortality instead shows constant widths of the intervals. Moreover, not all approaches appear to perform well in the winter season. The Statens Serum Institut (2020a), for instance, shows significant excess mortality in all winter seasons. As the excess mortalities are the differences between the numbers of observed and expected deaths, their forecast seems to be systematically misspecified for winter. Our model tries to account for these limitations of previous approaches and can, due to its cross-country perspective, be well implemented for a multipopulation analysis of excess mortality.

As the magnitude of our results does not permit us to report everything that could be derived from our model, we restrict the results to one dimension at a time (e.g., either demographics or geography by week). Indeed, we derive simulation results for all 152 variables. To illustrate the depth of our analysis, we have added the detailed results for all age groups in Spain as an example in Appendix E, since Spain is one of the larger countries in Europe and has witnessed significant mortality due to COVID-19. Moreover, the Spanish COVID-19 data and surveillance are of relatively high quality.

Our results show that there appears to be a general excess mortality caused by the COVID-19 pandemic, which affects different age groups and countries heterogeneously. We needed to combine the population below age 65 into a single group since the HMD data do not differ within the age group of 15–64 years and the number of deaths under age 15 has been too few for meaningful statistical analysis. This somewhat limits our understanding of the sensitivity of mortality in the different age groups. However, this issue did not bias our understanding of the overall excess mortality since the excess mortality for persons under 65 years of age was found to be rather limited. As our study was limited to countries with sufficiently long time series data, other countries that are affected strongly by the pandemic, such as Italy, are missing, which limits our conclusions to the countries analyzed here. The excess mortalities quantified here are not representative globally. The regional variations are at least partly explained by the differing courses of the epidemic as well as by the different NPIs that were implemented nationally or regionally during the study period (Ritchie et al., 2020). Thus, it is difficult to quantify the actual attributions of COVID-19 infections to the overall population mortality risks (Chaudry, Dranitsaris, Mubashir, Bartoszko, & Riazi, 2020; Hadjidemetriou, Sasidharan, Kouyialis, & Parlikad, 2020). According to IHME (2021), six different drivers influence excess mortality estimates. In addition to direct COVID-19 deaths, additional deaths caused by an overburdened health care system or mental health disorders may appear. On the other hand, the mobility and contact restrictions discussed earlier might have lowered the mortality that was due to traffic accidents and other infectious diseases. Finally, the deaths of frail individuals may have been moved forward, since these individuals, who would have died later due to their chronic conditions, have died earlier because of COVID-19. These cases would then be associated with a temporal spike in mortality and eventual death numbers that were below expectations. While we discussed some of these points qualitatively in our paper, our data do not allow us to include these points in the model, as this would require detailed cause-specific mortality data.

Mortality due to a specific disease can be addressed by the case fatality risk (CFR), which is the risk of death after infection but, however, is quite vulnerable to bias in outbreaks (Lipsitch et al., 2015). The international CFR estimates for COVID-19 are biased due to the demographic characteristics of the cases, time lags between the reporting of cases and deaths, underreporting of cases and deaths, and capacities of national healthcare systems, among other unobservable factors. Therefore, assessing the international differences in mortality due to COVID-19 without accounting for these characteristics and factors is inadvisable (Backhaus, 2020; Dudel et al., 2020; Vanella et al., 2021).

Our model only included temporal variables as predictors. The SARIMA models implicitly include unobservable trends by estimating the stochasticity of the mortality trends. However, in the baseline period, unobserved events are not included in the model. Mortality trends are, among others, influenced by infection activity, which depends on the contact rates among individuals (Kirkeby, Halasa, Gussmann, Toft, & Græsbøll, 2017). Many countries, therefore, implemented interventions during the pandemic that aimed at contact restrictions. As we have not witnessed similar occurrences in the past, we could not quantify the impacts of such measures in our model. We, therefore, decided to provide rather qualitative results for the excess mortalities that were observed in the study countries, along with the possible impact of contact restrictions. We purposely rejected reporting quantitative results on that, as we cannot do that in a responsible, statistical manner. Future studies might further address this topic.

The classical Lee-Carter model and its extensions, which usually perform exceptionally well in mortality forecasting, might not be applicable in their pure forms for the near future, as the long-term overall effect of COVID-19 on age-specific mortality patterns and its summary measures, such as life expectancy at birth and lifespan inequality, is yet unobserved. Therefore, the mortality trends derived from historical mortality data might not be completely representative of future trends. As we have not witnessed a similar pandemic in the near past, an adjustment factor to the classic Lee-Carter models could be appropriate, which would transform them to Bayesian models. The additional information, e.g., the mortality changes due to COVID-19, is difficult to assess; however, as the CFRs are biased, as has been discussed, the actual prevalence of the disease among the population is unknown. Many patients who experience only mild symptoms or are completely asymptomatic (Istituto Superiore di Sanità, 2020) will not be detected (Mizumoto, Kagaya, Zarebski, & Chowell, 2020). Moreover, the prevalence estimates are potentially influenced by the variations in COVID-19 countermeasures that are introduced by the different countries and even by subnational geographical units. By examining the number of deaths in Spain, as illustrated in Figure 7, we observe sharp mortality decreases after the peak in week 13, i.e., the last week of March. In mid-March, Spain introduced national countermeasures to contain the spread of the virus (Hogan Lovells Solutions, 2020), which presumably led to the mortality decrease after calendar week 13 and by considering the time lag of up to 2 weeks between the infection and death of a specific person (Vanella et al., 2021). As we lack an experimental environment for individual measures, which would be needed to estimate their effects on virus spread and mortality, the number of deaths that was prevented by countermeasures cannot be quantified. Our stochastic investigation illustrates the potential influence of pure stochasticity on the observed death numbers, which indicates that a deterministic inspection of the reported death numbers does not provide a reliable estimate of the impacts of COVID-19 countermeasures but simply a qualitative orientation. Therefore, the available data do not allow estimations of mortality levels under “normal circumstances”, i.e., if we had no active contact reduction measures. Our COVID-19-adjusted estimates have shown, however, that the excess mortality in calendar weeks 13 and 14 was exceptionally high, even after considering the reported COVID-19 deaths. This may be associated either with contemporaneous external factors that are not associated with the pandemic; to the indirect mortality effects of the pandemic, such as surplus mortality through causes due to reduced healthcare capacities in overwhelmed healthcare systems (Roberton et al., 2020); or due to bias in the COVID-19 death numbers (Backhaus, 2020) during that time. After the implementation of COVID-19 countermeasures in the study countries, our COVID-19 adjusted excess mortality estimates were temporarily significantly negative. The cause of this is unknown; however, the direct effects of NPIs on other infectious diseases (including influenza) as well as the indirect effects that decreased the disease burdens from other causes of death, such as air pollution (Contini & Costabile, 2020) or accidents (Shilling & Waetjen, 2020), are possible. Only cause-specific mortality estimates, including excess mortalities, would shine light on these effects; however, the majority of the countries included here do not provide causes of death in a sufficiently timely manner to allow this. In principle, however, the model presented here would allow stratification by death cause.

An experimental approach might adjust the mortality data to the prevalence rates of active cases among the population. As these are not available for all countries by similar measurements, i.e., in coherent demographic groups (Dudel et al., 2020), well-considered methods for adjusting the available data need to be applied. Making adjustments by using simulations derived from population-based seroprevalence studies might be one solution. As this appears to exceed the scope of our investigation, we will not elaborate on that further in this paper. Further studies could consider this, however, for mortality forecasting.

One final note regarding our methodology is that we rely on a mortality forecast model, which produces PIs that increase with the forecast horizon. As such, employing our model for a very long forecast horizon may hinder the detection of excess mortality. A 1-year forecast horizon, as we have employed in our paper, seems to be a balanced choice.

## Conclusions and outlook

The excess mortalities during an epidemic are commonly computed by using comparisons of observed death numbers or death rates to ex ante predictions of mortality. Based on an extension of the Lee-Carter mortality model (Lee & Carter, 1992; Vanella, 2017), we introduced a framework for including not only the abovementioned autocorrelations of the mortality time series and cross-correlations among the mortality time series into the analysis but also consider the long-term trends in the time series. We have considered these points in our model by using a combination of PCA, SARIMA models, and classic time series analysis. In particular, the inclusion of cross-country mortality correlations in the model appears to be a crucial aspect in times of pandemics due to the spread of the pathogen over international borders. We have covered the common mortality trends that are induced by the spread of the virus within our PCA. Moreover, our approach provides an efficient way to conduct multipopulation studies on mortality development. We have illustrated how the methods, which are established in demographic forecasting, can enrich the common epidemiological approaches that are employed in excess mortality studies. Our results identified significant differences in excess mortality among different subpopulations and countries, which could be investigated further. The detailed analysis of COVID-19-associated deaths against the deaths due to other causes has shown that the mortality levels due to other causes have been slightly below expectations in 2020 in our study countries. This suggests a positive effect of NPIs in preventing deaths due to other causes as well, not just COVID-19 related, and has been described in detail for other respiratory infections, such as influenza (Fricke, Glöckner, Dreier, & Lange, 2021). This, however, cannot be analyzed conclusively based on our available data.

Our application has illustrated the power of timely and detailed surveillance data on mortality trends to inform health policies in a timely manner and provides scientific support for decision-making. In addition to the case of all-cause mortality covered in this article, our approach could be applied to cause-specific mortality data. This would provide additional insights into the mortality patterns related to specific diseases, independent of the outbreak of an epidemic.

## Figures and Tables

**Fig. 11:**
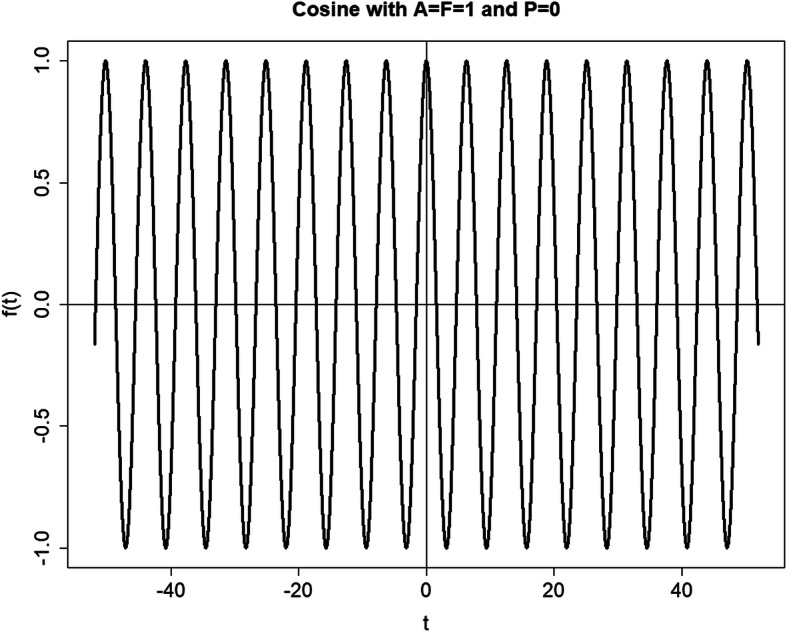
Cosine function with amplitude 1 and period 2*π*

**Fig. 12:**
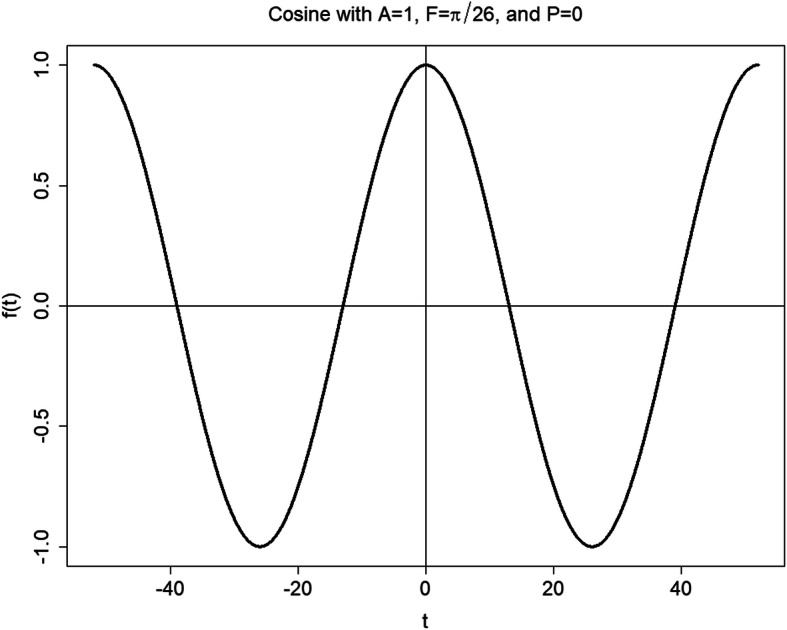
Cosine with amplitude 1 and period 52

**Fig. 13:**
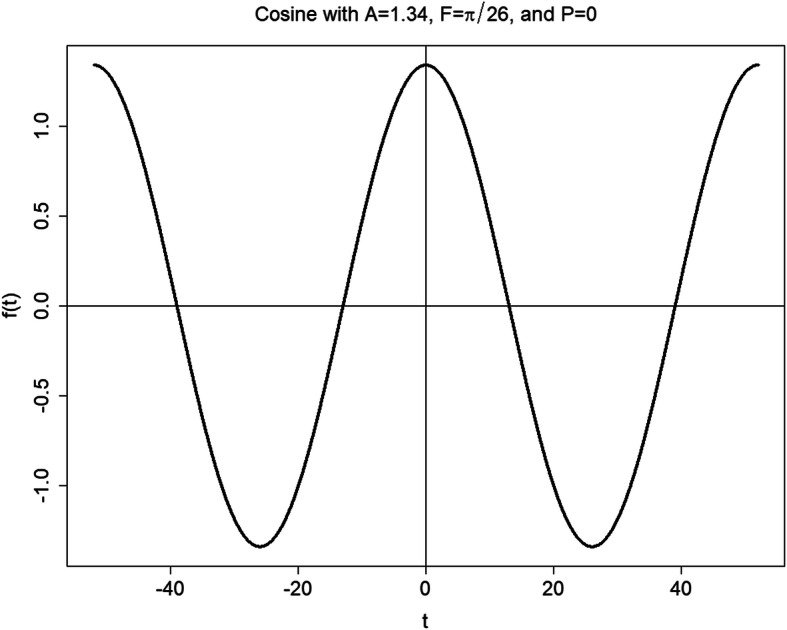
Cosine function with amplitude 1.34 and period 52

**Fig. 14:**
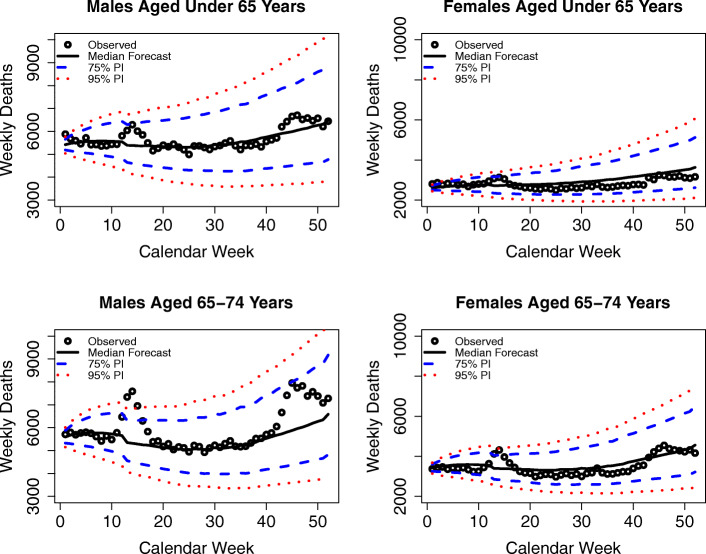
Observed and predicted weekly sex-specific deaths in 2020 for the 19 study countries and by age group below 75 years, Sources: Human Mortality Database (2021); Own computation and design

**Fig. 15:**
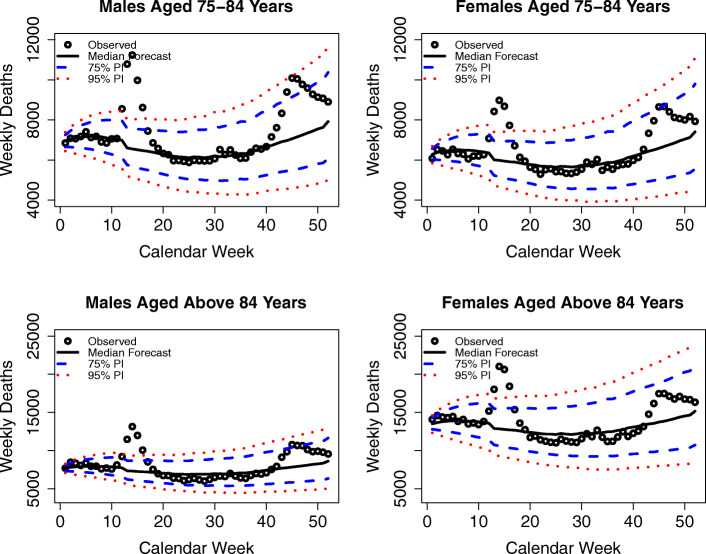
Observed and predicted weekly sex-specific deaths in 2020 for the 19 study countries and by age group above 74 years. Sources: Human Mortality Database (2021); Own computation and design

**Fig. 16:**
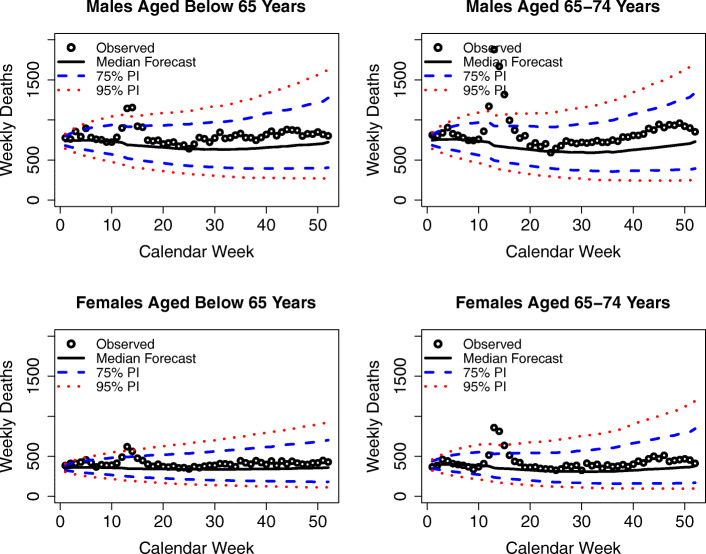
Observed and predicted weekly death numbers in 2020 in Spain by sex and age below 75 years of age. Sources: Human Mortality Database (2021); Computations and design by the authors

**Fig. 17:**
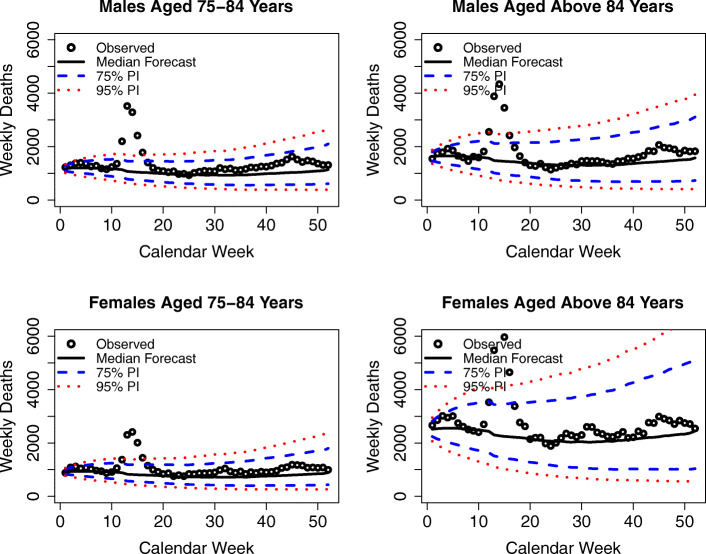
Observed and predicted weekly death numbers in 2020 in Spain by sex and age above 74 years of age. Sources: Human Mortality Database (2021); Computations and design by the authors

## Data Availability

The datasets used and/or analyzed during the current study are available from the corresponding author on reasonable request.

## Appendix A. Selected approaches for estimating excess mortality associated with COVID-19

**Table 2:** Details of the presented approaches for excess mortality estimation during the COVID-19 pandemic

**Study**	**Geography**	**Demographics**	**Data**	**Method**	**Main Results**	**Consideration of Long-term Trends in Mortality**	**Quantification of Stochasticity**	**Dealing with Correlations among time series**
Magnani et al.	4,433 Italian municipalities	No sex distinction Two age groups	Daily death numbers from January 1st to April 15th, 2015-2020 Yearly population estimates for January 1st, 2015–2019 Daily Death numbers attributed to COVID-19 by lab testing in 2020	Mean mortality rates by calendar days for 2015–2020 are computed Daily mortality rate estimates in 2020 are computed and assumed to follow a Poisson distribution Relative risk estimates with 95% CIs are computed relative to the baseline mean rates for 2015–2019	Statistically significant increase in mortality rates between early-March and mid-April, 2020 in Italy Only significant for North and parts of Central Italy Only significant for persons above age 59, except for Lombardia (both age groups significant)	None	Poisson assumption for observed deaths in 2020	None
Michelozzi et al.	19 Italian cities	Two sexes Four age groups	Daily death numbers from January 1st, 2015 to April 18th, 2020	Mean death numbers by calendar days for 2015–2020 are computed Daily death numbers implicitly assumed to be Gaussian, 95% PIs of baseline data are computed Comparison of observed death numbers with PIs	Statistically significant excess mortality in Italy between mid-March and mid-April, 2020 In the North, statistically significant excess mortality for males for all investigated age groups (age 15 and older), for females for age groups 65 and older In the Center and South only statistically significant excess mortality for very old females (above age 84) and males (above age 74)	None	Prediction assumed Gaussian with a standard deviation of past five years	None
NYC DOHMH	New York City	None	Daily death numbers with a lab-confirmed COVID-19 infection in 2020	Daily death numbers are predicted by OLS fit of the Serfling model to daily death numbers of baseline period Comparison of observed with expected daily death numbers Comparison of COVID-19 associated deaths with overall excess mortality estimate	Excess mortality after March 10th, with a large share of confirmed or suspected COVID-19 cases	Serfling model	None	None
EUROMOMO	22 EU-28 countries 2 German federal states	No sex distinction Seven age groups	Weekly all-cause death numbers since week 1, 2016	Poisson model fit to baseline data 95% PIs derived from the GLM model Comparison of observed weekly death numbers to 95% PIs	Statistically significant excess mortality in all countries for age groups 45+ between circa calendar weeks 11 and 19, 2020; death numbers above threshold in calendar weeks 13–15 for persons aged 15–44 years; no significant excess mortality among children Significant excess mortality observed in Belgium, France, Ireland, Italy, Netherlands, Portugal, Spain, Sweden, Switzerland, and the UK No significant excess mortality in Austria, Denmark, Estonia, Finland, Greece, Hungary, Luxembourg, Malta, Norway, and the two German federal states	Poisson model	PIs from the GLM model	None
Ours	19 countries in Europe and the Middle East	Two sexes Four age groups	Weekly all-cause mortality rate estimates since week 2, 2000 by HMD Daily official COVID-19 associated deaths in 2020 by ECDC	Principal component analysis on all 152 time series of logit mortality rates simultaneously OLS fit of logistic SARIMA model to past course of first PC Monte Carlo simulation of weekly forecasts of all PCs Retransformation of simulations to simulations of age-sex- and country-specific mortality rates Multiplication of simulations with population estimates for 2020 Derivation of nonparametric PIs for forecasts of death numbers Comparison of observed all-cause and COVID-19 associated weekly death numbers with PIs of forecasts	No significant excess mortality for persons below age 65, slightly significant excess mortality among females aged 65-74 years, statistically significant excess mortality among males above age 64 and females above age 74 years in calendar weeks 13–16, 2020 Statistically significant overall excess mortality only observable for Belgium, France, Netherlands, Scotland, Sweden, Poland, and Spain; results for Switzerland inconclusive; no significant excess mortality in Austria, Estonia, Finland, Hungary, Israel, Latvia, Lithuania, Norway, Portugal, Slovakia, and Slovenia	Logistic trend fitted to first principal component time series	SARIMA models and Monte Carlo simulation of principal component time series with nuisance derived from 20 years of past data, leading to stochastic estimates of all mortality rate series	Correlations among age groups and countries completely covered by principal component analysis

## Appendix B. Time series numbering

**Table 3:** Order of time series used in the analysis

Number	Country	Sex	Age group
1	Austria	Male	<65
2	Austria	Male	65–74
3	Austria	Male	75–84
4	Austria	Male	>84
5	Austria	Female	<65
6	Austria	Female	65–74
7	Austria	Female	75–84
8	Austria	Female	>84
9	Belgium	Male	<65
10	Belgium	Male	65–74
11	Belgium	Male	75–84
12	Belgium	Male	>84
13	Belgium	Female	<65
14	Belgium	Female	65–74
15	Belgium	Female	75–84
16	Belgium	Female	>84
17	Switzerland	Male	<65
18	Switzerland	Male	65–74
19	Switzerland	Male	75–84
20	Switzerland	Male	>84
21	Switzerland	Female	<65
22	Switzerland	Female	65–74
23	Switzerland	Female	75–84
24	Switzerland	Female	>84
25	Spain	Male	<65
26	Spain	Male	65–74
27	Spain	Male	75–84
28	Spain	Male	>84
29	Spain	Female	<65
30	Spain	Female	65–74
31	Spain	Female	75–84
32	Spain	Female	>84
33	Estonia	Male	<65
34	Estonia	Male	65–74
35	Estonia	Male	75–84
36	Estonia	Male	>84
37	Estonia	Female	<65
38	Estonia	Female	65–74
39	Estonia	Female	75–84
40	Estonia	Female	>84
41	Finland	Male	<65
42	Finland	Male	65–74
43	Finland	Male	75–84
44	Finland	Male	>84
45	Finland	Female	<65
46	Finland	Female	65–74
47	Finland	Female	75–84
48	Finland	Female	>84
49	France	Male	<65
50	France	Male	65–74
51	France	Male	75–84
52	France	Male	>84
53	France	Female	<65
54	France	Female	65–74
55	France	Female	75–84
56	France	Female	>84
57	Scotland	Male	<65
58	Scotland	Male	65–74
59	Scotland	Male	75–84
60	Scotland	Male	>84
61	Scotland	Female	<65
62	Scotland	Female	65–74
63	Scotland	Female	75–84
64	Scotland	Female	>84
65	Hungary	Male	<65
66	Hungary	Male	65–74
67	Hungary	Male	75–84
68	Hungary	Male	>84
69	Hungary	Female	<65
70	Hungary	Female	65–74
71	Hungary	Female	75–84
72	Hungary	Female	>84
73	Israel	Male	<65
74	Israel	Male	65–74
75	Israel	Male	75–84
76	Israel	Male	>84
77	Israel	Female	<65
78	Israel	Female	65–74
79	Israel	Female	75–84
80	Israel	Female	>84
81	Lithuania	Male	<65
82	Lithuania	Male	65–74
83	Lithuania	Male	75–84
84	Lithuania	Male	>84
85	Lithuania	Female	<65
86	Lithuania	Female	65–74
87	Lithuania	Female	75–84
88	Lithuania	Female	>84
89	Latvia	Male	<65
90	Latvia	Male	65–74
91	Latvia	Male	75–84
92	Latvia	Male	>84
93	Latvia	Female	<65
94	Latvia	Female	65–74
95	Latvia	Female	75–84
96	Latvia	Female	>84
97	Netherlands	Male	<65
98	Netherlands	Male	65–74
99	Netherlands	Male	75–84
100	Netherlands	Male	>84
101	Netherlands	Female	<65
102	Netherlands	Female	65–74
103	Netherlands	Female	75–84
104	Netherlands	Female	>84
105	Norway	Male	<65
106	Norway	Male	65–74
107	Norway	Male	75–84
108	Norway	Male	>84
109	Norway	Female	<65
110	Norway	Female	65–74
111	Norway	Female	75–84
112	Norway	Female	>84
113	Poland	Male	<65
114	Poland	Male	65–74
115	Poland	Male	75–84
116	Poland	Male	>84
117	Poland	Female	<65
118	Poland	Female	65–74
119	Poland	Female	75–84
120	Poland	Female	>84
121	Portugal	Male	<65
122	Portugal	Male	65–74
123	Portugal	Male	75–84
124	Portugal	Male	>84
125	Portugal	Female	<65
126	Portugal	Female	65–74
127	Portugal	Female	75–84
128	Portugal	Female	>84
129	Slovakia	Male	<65
130	Slovakia	Male	65–74
131	Slovakia	Male	75–84
132	Slovakia	Male	>84
133	Slovakia	Female	<65
134	Slovakia	Female	65–74
135	Slovakia	Female	75–84
136	Slovakia	Female	>84
137	Slovenia	Male	<65
138	Slovenia	Male	65–74
139	Slovenia	Male	75–84
140	Slovenia	Male	>84
141	Slovenia	Female	<65
142	Slovenia	Female	65–74
143	Slovenia	Female	75–84
144	Slovenia	Female	>84
145	Sweden	Male	<65
146	Sweden	Male	65–74
147	Sweden	Male	75–84
148	Sweden	Male	>84
149	Sweden	Female	<65
150	Sweden	Female	65–74
151	Sweden	Female	75–84
152	Sweden	Female	>84

## Appendix C. Basics of cosine functions

A cosine function can be characterized by its amplitude *A*, its periodicity or frequency *F*, and its phase angle *P* (Fuller, 1996):

$$ f(t)= Acos\left(F\ast t+P\right) $$

The simplest form of a cosine takes the values *A* = *F* = 1, *P* = 0. Graphically, this function can be illustrated as follows.

A period of $\frac{\pi }{26}$ will now stretch the curve horizontally by the reciprocal.

An amplitude of 1.34, as estimated by OLS in model 1 of our study, leads to a vertical stretch in the curve, as illustrated in Figure 13.

## Appendix D. Excess mortality estimates by demographic groups

Here, we report more detailed results that are generated by our model by demographic groups for our 19 study countries for 2020. We observe distinct differences in excess mortality between the age groups and two genders.

Both sexes and all age groups showed peaks in the number of deaths between weeks 12 and 17 and after calendar week 40 of 2020. However, a more detailed analysis shows increases beyond the upper limits of the 95% PIs only for the very old age groups and in spring 2020. For persons aged 75 and above, we observe a significant increase in mortality for weeks 13–16. For the 65–74 age group, the increase was statistically significant for males only. The mortality increases for persons below age 65 since the COVID-19 crisis are not statistically significant for either sex.

## Appendix E. Detailed model results for Spain

For illustrative purposes, we show the complete results for one of the countries that were analyzed in our study. Figures 16 and 17 show the observed and predicted weekly deaths by age group and sex in Spain in 2020. Statistically significant excess mortality is observable in all panels.

## Appendix F. Shares of variance explained by the principal components

**Table 4:** Shares of variance explained by all logit-mortality rate time series

Principal component no.	Explained variance [in %]	Explained variance cumulative [in %]
1	54.9	54.9
2	5.8	60.7
3	2.6	63.3
4	1.8	65.1
5	1.6	66.7
6	1.4	68.1
7	1.1	69.2
8	1.1	70.2
9–152	<1	100

## Abbreviations

AIC: Akaike’s information criterion
ARIMA: Autoregressive integrated moving average
BIC: Bayesian information criterion
CDC: Centers for Disease Control and Prevention
CFR: Case fatality risk
CI: Confidence interval
cos: Cosine
DOHMH: New York City Department of Health and Mental Hygiene
ECDC: European Centre for Disease Prevention and Control
e.g.: Exempli gratia
EUROMOMO: European mortality monitoring
exp(x): Euler’s number to the power of x
GLM: Generalized linear model
HMD: Human mortality database
i.e.: id est
IHME: Institute for Health Metrics and Evaluation
*λ*_*i*_: *i*th loading
*μ*_*i*, *τ*_: *i*th logit mortality rate estimate for week *τ*
MCMC: Markov Chain Monte Carlo
NPI: Nonpharmaceutical intervention
PC: Principal component
PCA: Principal component analysis
PI: Prediction interval
**Π**_*t*_: Simulation matrix of the PCs in period *t*
SARIMA: Seasonal autoregressive integrated moving average
US: United States of America
*w*: Week
WASCSMR: Weekly age-, sex-, and country-specific mortality rate

## Footnotes

1 Census estimates.

2 Pneumonia or influenza, cardiovascular or renal, and others.

3 See Box et al. (2016) or Shumway and Stoffer (2016) for detailed presentations of ARIMA models.

4 With the exceptions of Luxembourg and Iceland, whose relatively small populations and death numbers do not allow derivation of representative weekly estimates.

5 Scotland does not offer data for week 1, 2000. To include Scotland in the analysis, we start in week 2 for all countries.

6 1039 weeks in the rows, 4 age groups times 2 sexes times 19 countries in the columns.

7 Note that OLS and MLE lead to the same estimates under the Gauß-Markov assumptions. OLS, however, allows for computation of the R² value.

8 Appendix F provides the shares of the variance in the original variables that are explained by the PCs.

9 E.g., PC2 covers the cohort shifts within age groups. For long-term forecasts, the PC should therefore be modeled in more detail. Within the scope of our paper, which is an investigation of the short-term fluctuations in mortality, the random walk assumption is sufficient.

10 Excluding Scotland, as there are no weekly COVID-19-specific time series data on mortality available.
